# Dual-energy X-ray absorptiometry and adiposity index are sensitive methods to evaluate central fat accumulation in women with polycystic ovary syndrome and normal body mass index

**DOI:** 10.20945/2359-3997000000627

**Published:** 2023-05-29

**Authors:** Gislaine Satyko Kogure, Victor Barbosa Ribeiro, Maria Célia Mendes, Rui Alberto Ferriani, Cristiana Libardi Miranda Furtado, Rosana Maria dos Reis

**Affiliations:** 1 Universidade de São Paulo Faculdade de Medicina de Ribeirão Preto Departamento de Ginecologia e Obstetrícia Ribeirão Preto SP Brasil Setor de Reprodução Humana, Departamento de Ginecologia e Obstetrícia, Faculdade de Medicina de Ribeirão Preto, Universidade de São Paulo, Ribeirão Preto, SP, Brasil; 2 Instituto Federal de São Paulo Jacareí SP Brasil Instituto Federal de São Paulo, campus Jacareí, Jacareí, SP, Brasil; 3 Universidade Federal do Ceará Centro de Pesquisa e Desenvolvimento de Medicamentos Programa de Pós-graduação em Ciências Médicas e Cirúrgicas Fortaleza CE Brasil Centro de Pesquisa e Desenvolvimento de Medicamentos, Programa de Pós-graduação em Ciências Médicas e Cirúrgicas, Universidade Federal do Ceará, Fortaleza, CE, Brasil

**Keywords:** Dual-energy X-ray absorptiometry, body mass index, central obesity, polycystic ovary syndrome

## Abstract

**Objective:**

This study aimed to determine the differences in body fat distribution and central obesity indicators using dual-energy X-ray absorptiometry (DXA), adiposity indices, and anthropometric indices between women with and without polycystic ovary syndrome (PCOS).

**Materials and methods:**

Clinical and laboratory examination history, including transvaginal ultrasound, fasting blood samples, anthropometric measurements, and DXA scans were conducted in 179 women with PCOS (PCOS group) and 100 without PCOS (non-PCOS group). The volunteers were grouped by body mass index (BMI): normal (18-24.9 kg/m^2^), overweight (25-29.9 kg/m^2^), or obese (>30 kg/m^2^). The visceral adiposity index (VAI) and lipid accumulation product (LAP) were calculated, regions of interest (ROIs) were determined, and the fat mass index (FMI) was calculated using DXA.

**Results:**

VAI, LAP, ROIs, FMI, and adiposity indices by DXA were higher in women with PCOS and normal BMI. In both PCOS and non-PCOS groups, the ROIs progressively increased from normal BMI to overweight and obese, and from overweight to obese. Obese women with PCOS showed high trunk fat mass. However, obesity was not able to modify these trunk/periphery fat ratios in PCOS from overweight to higher BMI. These variables were associated with the incidence of PCOS.

**Conclusion:**

In women with PCOS and normal BMI, both DXA and the adiposity indices, VAI and LAP, are more sensitive methods to evaluate total body fat and fat accumulation in the central abdominal region. It was also observed that as BMI increased, the differences in measurements between women with and without PCOS decreased.

## INTRODUCTION

Polycystic ovary syndrome (PCOS) is a common endocrine disorder in women of reproductive age, with an estimated prevalence of 6%-20% (1). Ovulatory dysfunction and impaired fertility are common reproductive outcomes related to PCOS pathophysiology, possibly triggered by androgen excess. Beyond hyperandrogenism, several clinical implications include metabolic syndrome, insulin resistance (IR), abnormal glucose metabolism, dyslipidemia with increased risk of type 2 diabetes mellitus, cardiovascular disease (CVD), and psychological disturbances (2). The prevalence of obesity in PCOS is reportedly 30%-70% (3). However, a small proportion of patients present with a normal body mass index (BMI; ≤25 kg/m^2^) which can minimize the adverse effects of syndrome-related hormonal and metabolic profiles, except IR (4).

Central obesity plays an important role in PCOS development and is associated with metabolic disturbances and IR, regardless of normal BMI, overweight, or obesity (5,6). Different methods have been used to evaluate body fat distribution. Whole-body dual-energy X-ray absorptiometry (DXA) scans allow estimation of the total, abdominal, and extremity fat mass (7). Among double indirect procedures, anthropometry is a simple, non-invasive, and low-cost method that can reach more participants (8). Other indicators that consider fat accumulation and distribution are the lipid accumulation product (LAP), an index to measure the excessive accumulation of abdominal fat based on the triglyceride (TG) level and waist circumference (WC) (9), and the visceral adiposity index (VAI), an index to assess fat distribution and function using WC, BMI, TG, and high-density lipoprotein cholesterol (HDL-C) levels (10).

Considering the importance of evaluating central obesity and its relationship with metabolic disorders and CVD risk, it is necessary to document BMI and estimate central obesity to better stratify the risk of PCOS in women. This study aimed to determine the differences in body fat distribution and central obesity indicators using DXA, adiposity, and anthropometric indices between BMI-matched women with and without PCOS, as well as in the metabolic and hormonal features of the disorder.

## MATERIALS AND METHODS

### Study design and participants

This was an observational, retrospective study that evaluated data from women with PCOS and control samples with regular menstrual cycles recruited at the Human Reproduction Service of the Gynecology and Obstetrics Department of Ribeirão Preto Medical School, University of São Paulo. Data from two clinical trial studies were evaluated, including a non-randomized clinical trial (NRCT) with sample collection between 2010 and 2013 and an RCT conducted between 2014 and 2016. All related trials for these interventions were approved by the Brazilian Clinical Trials Registry (NRCT, RBR-7p23c3) and the International Standard Randomized Controlled Trial Registry (RCT, ISRCTN10416750). This study was approved by the institutional review board of the University Hospital (HC-FMRP-USP – CAAE: 14496719.4.0000.5440). This study included 279 women aged 18-39 years: 179 women with PCOS (PCOS group), and 100 women with ovulatory menstrual cycles (non-PCOS group), with normal (18-24.9 kg/m^2^), overweight (25-29.9 kg/m^2^), or obese (>30 kg/m^2^) BMIs according to the World Health Organization criteria. All participants provided written informed consent.

Women with PCOS were diagnosed according to the Rotterdam ESHRE/ASRM PCOS Consensus (11) based on the presence of at least two out of three criteria: clinical and/or biochemical hyperandrogenism, oligo/anovulation, and polycystic ovaries on ultrasonography. The non-PCOS group comprised healthy women with regular menstrual cycles of 24-32 days, with 3-7 days of duration and testosterone levels and free androgen index (FAI) within the reference interval and those without hirsutism. Women with and without PCOS were included irrespective of race, parity, or social class. Participants with incomplete data; users of drugs – if within 6 months prior of enrollment to the study – that interfered with the hypothalamic-pituitary hormone function (i.e., hormonal contraceptives, estrogen and progestin drugs, hormone therapy, gonadotropin-releasing hormone analogs and antagonists); pregnant women; smokers; and women with congenital adrenal hyperplasia, thyroid diseases, hyperprolactinemia, or Cushing's syndrome were excluded from the study.

### Clinical and biochemical parameters

Transvaginal pelvic ultrasonography was performed using a Voluson 730 Expert instrument (GE Medical Systems, Zipf, Austria) to evaluate the presence of polycystic ovaries. Fasting blood samples were drawn until the eighth day of the menstrual cycle (early follicular phase) or any day when the participant experienced amenorrhea. Blood tests included androgens (total testosterone, androstenedione), sex hormone-binding globulin (SHBG), fasting insulin, glucose, and fasting lipid profiles.

Fasting insulin and SHBG levels were measured using a chemiluminescence method (Immulite® 2000 Immunoassay System; Siemens®, Santa Ana, CA, USA). Fasting blood glucose was determined using the oxidase method (CMD 800X1/CMD 800iX1, Wiener Lab, São Paulo, Brazil). IR was quantified using the homeostatic model assessment (HOMA-IR): (fasting blood glucose in mg/dL ×0.05551) × fasting insulin in μUI/mL/22.5 (12). Plasma total cholesterol (TC), HDL-C, and TG levels were measured using an enzymatic method (CMD 800X1/CMD 800iX1, Wiener Lab, São Paulo, Brazil). Low-density lipoprotein cholesterol (LDL-C) levels were measured using the Friedewald formula: LDL-C = TC – (HDL-C+TG/5) (13). Testosterone and androstenedione levels were measured using a radioimmunoassay in the NRCT (Immulite1000 Immunoassay System; Siemens®, Santa Ana, CA, USA) and chemiluminescence method in the RCT (Immulite 1000; Immunoassay System; Siemens®) – Study 2). The FAI was calculated as total testosterone (nmol/L)/SHBG (nmol/L) × 100] (14).

### Anthropometric measurements

Height was recorded to the nearest 0.1 cm using a standard anthropometer, and weight to the nearest 0.5 kg using a weight scale (Filizola, São Paulo, Brazil). A non-elastic flexible measuring tape was used to measure, with all measurements taken by a single evaluator and recorded to the nearest 0.1 cm. WC was measured as the mid-distance between the lower ribs and iliac crest, and the hip circumference (HipC) was measured around the greatest circumference of the gluteal region. The following anthropometric indices were calculated: BMI (kg/m^2^), calculated by dividing body weight by the square of the height; waist-to-hip ratio (WHR), calculated by dividing WC (cm) by HipC (cm), and waist-to-height ratio (WHtR), calculated by dividing WC (cm) by height (cm).

### Adiposity indices – VAI and LAP

VAI was measured using the formula [WC/36.58 + (1.89×BMI) * (TG/0.81) * (1.52/HDL)] and LAP was measured using [{WC (cm) - 58} *TG (nmol/L)] (15).

### DXA scans

DXA in whole-body array mode (Hologic device Discovery® QDR Series, Waltham, MA) was used to measure whole-body fat mass (BFM) and to differentiate between peripheral (legs and arms) and central fat mass. Default software readings were used to divide the body into six compartments: the head, trunk, arms, and legs. The scan was performed on all volunteers in morning after a 12-hour fast. All volunteers were instructed to wear comfortable, loose-fitting clothes avoidance of metal components. The regions of interest (ROIs) for the assessment of fat distribution were the abdominal trunk and android regions, and the following variables of fat distribution were calculated: total BFM (Total_FM_) (g), total body fat percentage (Total_%FM_), trunk BFM (Trunk_FM_) (g), trunk body fat percentage (Trunk_%FM_), android fat mass (And_FM_) (g), android fat percentage (_%FM_), gynoid fat mass (Gyn_FM_) (g), and gynoid fat percentage (Gyn_%FM_). The total fat mass index (FMI kg/m^2^) – Total_FM_ (kg)/height^2^ (m^2^), and other indices were calculated using the 5 Discovery Wi model software (S/N 84826) version 13.0, provided by the manufacturer (Waltham, MA), as follows: android/gynoid percentage fat ratio (A/G_RATIO_), trunk/leg fat percentage ratio (Trunk/Legs_%FAT_), and trunk/limb fat mass ratio (Trunk/Limb_FAT_). Limb fat was calculated as the total fat in arms and legs (g). In the upper arms, the limb fat included subcutaneous adipose tissue from the shoulder to the wrist. In the lower extremities, limb fat included subcutaneous adipose tissue from the hip to the ankle.

### Statistical analyses

All statistical analyses were performed using the PROC MIXED method of SAS, version 9.3 (SAS Institute Inc., University of North Carolina, NC, USA). Exploratory analysis of the data was performed using measures of the central position and dispersion. Chi-square tests were used to determine the associations between categorical variables, and t-tests and one-way analysis of variance were used to analyze continuous variables. A generalized linear model multivariate was used to investigate the linear relationships between the explanatory variables age, HOMA-IR, and FAI, and the dependent variables were the markers of fat mass. Statistical significance was set at *P* < 0.05.

## RESULTS

The clinical characteristics of the PCOS and non-PCOS groups are shown in Table 1. Levels of prolactin (PCOS 14.83 ± 11.38 ng/mL *vs* non-PCOS 14.98 ± 10.77 ng/mL; *P* = 0.92), 17-Hydroxy progesterone (PCOS 99.31 ± 60.50 ng/dL *vs* non-PCOS 101.75 ± 67.09 ng/dL, *P* = 0.79), and TSH (PCOS 2.33 ± 1.38 uIU/mL *vs* non-PCOS 2.24 ± 1.17 uIU/mL, *P* = 0.58) did not differ between groups. The PCOS group had higher testosterone and fasting insulin levels and FAI and HOMA-IR scores than the non-PCOS group. Moreover, the PCOS group had worse serum levels of TG, HDL-C, and SHBG. Overall, BMI and weight were increased in women with PCOS (*P* = 0.02 and *P* < 0.01, respectively), and in all markers of BFM and central obesity indicators (*P* < 0.05, for all), except for region gynoid by DXA (*P* = 0.08) and HipC by anthropometry (*P* = 0.30). In the PCOS group, 74 (41.34%) women were obese, 54 (30.17%) were overweight, and 51 (28.49%) were of normal weight. In the non-PCOS group, 33 (33.00%) were obese, 25 (25.00%) were overweight, and 52 (52.00%) had normal BMI values.

**Table 1:** Clinical characteristics evaluated in the PCOS and non-PCOS groups

Variables		PCOS (n = 179)	non-PCOS (n = 100)	*P* Value
		Mean (SD)	Mean (SD)	
Age (years)		28.41 (5.28)	29.37 (4.97)	0.13
Hormonal parameters				
	Total testosterone (ng/dL)	98.47 (45.43)	70.76 (29.43)	0.00
	FAI	9.46 (7.38)	5.26 (4.11)	0.00
	Androstenedione (ng/dL)	92.68 (50.28)	100.60 (34.93)	0.19
	SHBG (nmol/L)	50.78 (34.70)	62.64 (36.78)	0.01
	E2 (pg/mL)	72.85 (61.62)	135.52 (83.89)	0.00
	FSH (uUI/mL)	5.03 (2.40)	4.97 (5.76)	0.90
	LH (uUI/mL)	7.83 (6.06)	5.45 (5.94)	0.03
Metabolic parameters				
	Fasting glycaemia (mg/dL)	90.05 (17.15)	96.73 (20.96)	0.00
	Fasting insulin (mg/dL)	10.95 (10.40)	5.86 (5.13)	0.00
	HOMA-IR	2.53 (3.12)	1.32 (1.32)	0.00
	Total cholesterol (mg/dL)	190.66 (38.00)	193.88 (43.30)	0.54
	Triglycerides (mg/dL)	125.07 (96.36)	100.90 (55.19)	0.02
	HDL-C (mg/dL)	49.41 (11.31)	55.21 (14.93)	0.00
	LDL-C (mg/dL)	116.00 (34.47)	119.91 (34.91)	0.38
Anthropometric measurements				
	Weight (kg)	75.73 (16.50)	70.94 (16.69)	0.02
	Height (m)	1.61 (0.06)	1.62 (0.07)	0.30
	BMI (kg/m^2^)	29.00 (5.75)	27.05 (5.91)	0.00
	Waist circumference (cm)	86.73 (13.20)	78.75 (12.74)	0.00
	Hip circumference (cm)	106.96 (10.56)	105.54 (11.00)	0.30
	Waist-to-hip ratio	0.81 (0.08)	0.74 (0.07)	0.00
	Waist-to-height ratio	0.53 (0.10)	0.49 (0.08)	0.00
Adiposity Index				
	VAI	7.54 (5.34)	5.63 (2.59)	0.01
	LAP	44.10 (44.24)	27.50 (30.70)	0.00
Body fat mass – DXA				
	Total_FM_ (g)	30282.47 (9367.52)	26714.74 (10106.57)	0.00
	Total_%FM_	40.35 (5.04)	37.87 (7.08)	0.01
	Trunk_FM_ (g)	14084.29 (5000.51)	11274.17 (4907.06)	0.00
	Trunk_%FM_	39.05 (6.27)	34.47 (8.23)	0.00
	Android_FM_ (g)	2488.34 (1048.31)	1987.04 (1014.31)	0.00
	Android_%FM_	42.27 (6.90)	38.09 (9.54)	0.00
	Gynoid_FM_ (g)	5338.69 (1549.94)	4977.38 (1597.16)	0.08
	Gynoid_%FM_	44.21 (4.93)	43.21 (6.83)	0.19
Fat mass index – DXA				
	FMI (kg/m^2^)	11.87 (4.48)	10.35 (3.72)	0.00
	A/G_Ratio_	0.96 (0.14)	0.87 (0.15)	0.00
	Trunk/Legs_%FAT_	0.90 (0.15)	0.79 (0.14)	0.00
	Trunk/Limb_FAT_	0.93 (0.21)	0.77 (0.17)	0.00

P < 0.05. The data are presented in mean and (SD) standard deviation. PCOS: polycystic ovary syndrome; %: percentage; FM: fat mass; SHBG: sex hormone binding globulin; FAI: free testosterone index; E2: estradiol; LH: luteinizing hormone; FSH: follicle stimulating hormone; HOMA-IR: homeostatic model assessment; HDL-C: high density lipoproteins cholesterol; LDL: low density lipoproteins cholesterol; BMI: body mass index; VAI: visceral adiposity index; LAP: lipid accumulation product; And: android; Gyn: gynoid; FMI: total fat mass index; A/G ratio: android/gynoid fat ratio; Trunk/Limb_FAT_: trunk/limb fat mass ratio; Trunk/Legs_%FAT_: trunk/legs fat percentage ratio.

### Intergroup analysis

**Figure 1:**
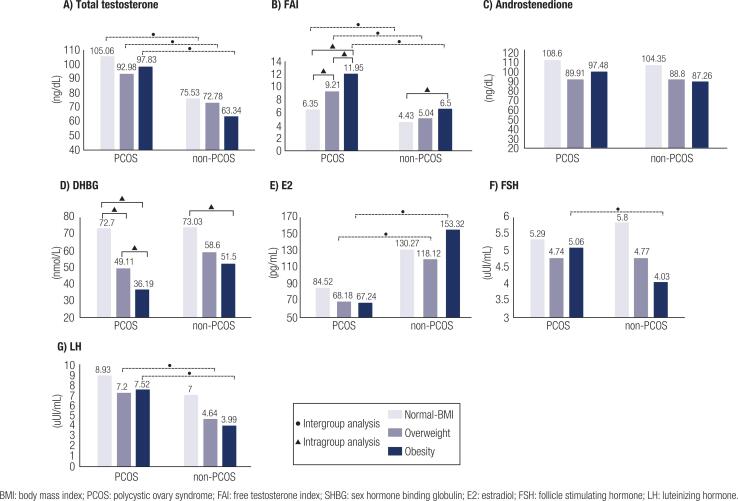
Intergroup and Intragroup analysis of hormonal parameters.

Compared to the non-PCOS group, the PCOS group showed increased testosterone levels and FAI scores in normal BMI (*P* < 0.001 and *P* = 0.027, respectively), overweight (*P* = 0.048 and *P* = 0.037, respectively), and obesity (*P* < 0.001 and *P* = 0.001, respectively) and fasting insulin level and HOMA scores in normal BMI (*P* = 0.007 and *P* = 0.010, respectively), overweight (*P* = 0.000 and *P* = 0.003, respectively), and obesity (*P* = 0.002 and *P* = 0.004, respectively) (Figures 1 [a-g] and 2 [a-g]).

**Figure 2:**
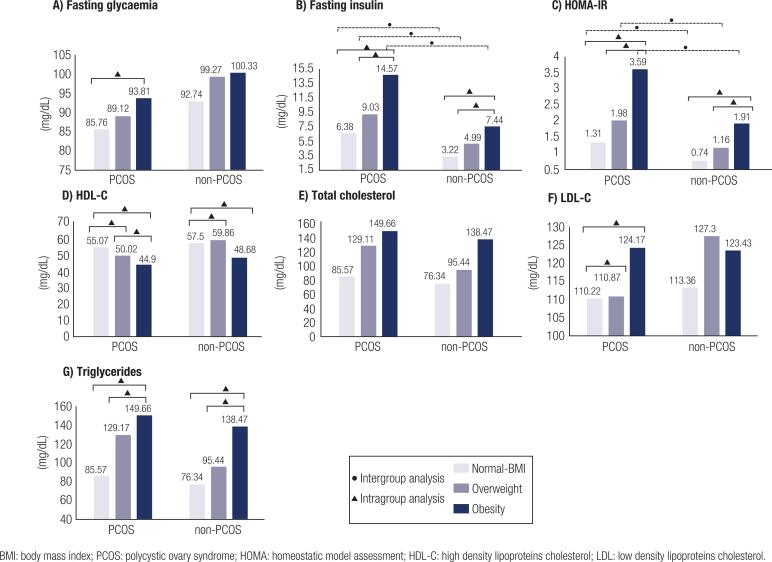
Intergroup and Intragroup analysis of metabolic parameters.

In the anthropometric indices, the PCOS group showed elevated values of WC and WHR in normal BMI (*P* < 0.001, both), overweight (*P* < 0.001, both), and obesity (*P* = 0.002, both), and WHtR in normal BMI and overweight (*P* < 0.001, both). The adiposity indexes, VAI, and LAP values were higher in women with PCOS with normal BMI (*P* = 0.003 and *P* < 0.001, respectively). The ROIs, FMI, and adiposity indices by DXA, the PCOS group showed elevated values in normal BMI in Total_FM_, Total_%FAT_, Trunk_FM_, Trunk_%FAT_, And_FM_, And_%FAT_, Gyn_%FAT_, FMI, A/G_RATIO_, Trunk/Legs_%FAT_, Trunk/Limb_FAT_; overweight in Trunk_FM_, Trunk_%FAT_, And_FM_, A/G_RATIO_, Trunk/Legs_%FAT_, Trunk/Limb_FAT_ and obesity in Trunk_%FAT_ and Trunk/Legs_%FAT_ (Table 2).

**Table 2:** Anthropometric indices, adiposity Index, body fat mass and fat mass index between PCOS and non-PCOS groups

		Normal BMI (kg/m^2^)				Overweight			Obesity	
		non-PCOS Mean (SD)	PCOS Mean (SD)	*P* Value	non-PCOS Mean (SD)	PCOS Mean (SD)	*P* Value	non-PCOS Mean (SD)	PCOS Mean (SD)	*P* Value
Anthropometric measurements										
	Weight (kg)	56.01 (5.56)	58.03 (5.84)	0.058	70.64 (5.85)	70.96 (7.64)	0.853	90.16 (11.05)	91.22 (11.19)	0.651
	Height (m)	1.61 (0.58)	1.62 (0.59)	0.784	1.62 (0.09)	1.60 (0.06)	0.205	1.62 (0.06)	1.61 (0.06)	0.477
	BMI (kg/m^2^)	21.45 (1.94)	22.18 (1.47)	0.461	27.19 (1.35)	27.40 (1.49)	0.549	34.07 (3.11)	34.86 (2.90)	0.205
	Waist circumference (cm)	67.27 (4.33)	72.37 (5.83)	0.000	77.71 (5.10)	83.84 (6.35)	0.000	93.58 (7.40)	99.06 (8.31)	0.002
	Hip circumference (cm)	95.15 (4.93)	96.06 (5.02)	0.397	107.74 (4.91)	105.24 (6.30)	0.095	116.64 (7.15)	115.95 (7.71)	0.670
	Waist-to-hip ratio	0.70 (0.04)	0.75 (0.05)	0.000	0.72 (0.05)	0.79 (0.06)	0.000	0.80 (0.06)	0.85 (0.07)	0.002
	Waist-to-height ratio	0.41 (0.03)	0.44 (0.03)	0.000	0.48 (0.03)	0.52 (0.03)	0.000	0.57 (0.04)	0.61 (0.04)	0.000
Adiposity Index										
	VAI	4.19 (1.03)	5.06 (1.68)	0.003	5.22 (1.55)	7.66 (7.61)	0.140	8.01 (3.10)	9.18 (4.15)	0.177
	LAP	7.60 (4.54)	14.18 (8.80)	0.000	19.46 (13.44)	39.16 (45.76)	0.052	58.85 (35.01)	69.50 (43.89)	0.257
Body fat mass – DXA										
	Total_FM_ (g)	17860.64 (4714.98)	20816.17 (3258.86)	0.001	27688.06 (6173.13)	27853.42 (5945.38)	0.912	38075.82 (5750.43)	38646.12 (6541.69)	0.697
	Total_%FM_	33.13 (5.02)	35.98 (4.02)	0.004	39.39 (7.56)	40.62 (4.17)	0.361	43.05 (4.47)	43.26 (4.02)	0.825
	Trunk_FM_ (g)	7058.48 (1736.94)	8555.91 (1867.54)	0.000	11346.34 (3116.69)	13257.99 (2364.86)	0.004	17044.65 (2912.40)	18646.94 (3402.59)	0.036
	Trunk_%FM_	28.60 (5.47)	32.70 (5.38)	0.001	35.58 (8.55)	39.86 (4.28)	0.005	41.57 (4.21)	42.99 (4.23)	0.145
	Android_FM_ (g)	1086.94 (348.62)	1411.90 (368.44)	0.000	1965.13 (599.01)	2238.06 (460.78)	0.035	3148.85 (623.20)	3480.92 (776.55)	0.055
	Android_%FM_	30.48 (6.47)	35.50 (5.51)	0.000	40.00 (9.35)	42.97 (5.18)	0.082	46.06 (4.34)	46.79 (4.69)	0.494
	Gynoid_FM_ (g)	3681.39 (768.48)	3944.88 (852.38)	0.157	5287.22 (1372.58)	5137.47 (1122.32)	0.622	6348.19 (1261.56)	6528.97 (1287.58)	0.544
	Gynoid_%FM_	40.30 (4.97)	42.47 (3.80)	0.027	44.94 (8.99)	44.69 (5.42)	0.882	45.38 (5.46)	45.14 (5.01)	0.834
Fat mass index – DXA										
	FMI (kg/m^2^)	7.03 (1.55)	7.87 (1.19)	0.007	10.45 (2.26)	11.75 (5.55)	0.286	14.48 (2.22)	14.95 (2.16)	0.357
	A/G_Ratio_	0.75 (0.10)	0.84 (0.13)	0.001	0.87 (0.11)	0.97 (0.10)	0.001	1.02 (0.09)	1.04 (0.10)	0.294
	Trunk/Legs_%FAT_	0.70 (0.08)	0.78 (0.14)	0.003	0.77 (0.16)	0.92 (0.12)	0.000	0.91 (0.10)	0.97 (0.11)	0.033
	Trunk/Limb_FAT_	0.69 (0.10)	0.78 (0.17)	0.002	0.74 (0.19)	0.97 (0.19)	0.000	0.89 (0.15)	1.01 (0.20)	0.002

P < 0.05. The data are presented in mean and (SD) standard deviation. PCOS: polycystic ovary syndrome; %: percentage; FM: fat mass; BMI, body mass index; VAI: visceral adiposity index; LAP: lipid accumulation product; And: android; Gyn: gynoid; FMI: total fat mass index; A/G ratio: android/gynoid fat ratio; Trunk/Limb_FAT_: trunk/limb fat mass ratio; Trunk/Legs_%FAT_: trunk/legs fat percentage ratio.

### Intragroup analysis

According to BMI, the PCOS group showed an increase in FAI scores and a decreasing SHBG level, progressively from normal BMI to overweight (P = 0.041 and P < 0.001, respectively), from normal BMI to obese (P < 0.001, both), and overweight to obese (P = 0.036 and P = 0.026, respectively), with significant differences between them (P < 0.001, both). In the non-PCOS group, the FAI scores increased from normal to obese (P = 0.019). Fasting insulin levels and HOMA score increased as BMI increased, from normal BMI to obese and overweight to obese in both groups. Fasting glycemia levels were elevated from normal BMI to obese, only in the PCOS group (P = 0.011) (Figures 1 [a-g] and 2 [a-g]).

The WC, HipC, and WHtR showed progressive increases with differences found from normal BMI to overweight and obese, and from overweight to obese (*P <* 0.001, all), in both PCOS and non-PCOS groups, as well as between them (*P <* 0.01, all). In the non-PCOS group, WHR did not differ from normal BMI to overweight (non-PCOS, *P* = 0.309). In both PCOS and non-PCOS groups, the ROIs (Total_FM_, Total_%FAT_, Trunk_FM_, Trunk_%FAT_, And_FM_, And_%FAT_, and Gyn_FM_) and LAP progressively increased with differences found from normal BMI to overweight and obese, and from overweight to obese, as well as between them (*P* < 0.001, all). However, in both groups, Gyn_%FAT_ did not differ from overweight to obese. In adiposity indices measured by DXA (FMI, A/G_RATIO_) progressively increased with differences found from normal BMI to overweight and obese, and from overweight to obese, as well as between them (*P* < 0.01, all). VAI, Trunk/Legs_%FAT_, and Trunk/Limb_FAT_ were not different between overweight and obese individuals (*P* = 0.102; *P* = 0.060; *P* = 0.189, respectively) in the PCOS group, and in the VAI and Trunk/Limb_FAT_ from normal BMI to overweight (*P* = 0.060 and *P* = 0.248, respectively) (Table 3).

**Table 3:** Differences in body fat mass and the central obesity indicators between normal BMI, overweight and obese in women with PCOS and non-PCOS

Variables					PCOS							non-PCOS			
		Sum of square	df	*F* value	Pr > *F*	1-2	1-3	2-3	Sum of square	df	*F* value	Pr > *F*	1-2	1-3	2-3
Anthropometric measurements															
	Weight (kg)	34493.427	2	217.440	0.000	0.000	0.000	0.000	21564.65	2	174.28	0.000	0.000	0.000	0.000
	Height (m)	.005	2	0.556	0.575	0.308	0.448	0.728	0.00	2	0.33	0.722	0.450	0.574	0.820
	BMI (kg/m^2^)	5049.835	2	526.774	0.000	0.000	0.000	0.000	2945.05	2	281.04	0.000	0.000	0.000	0.000
	Waist circumference (cm)	21914.012	2	217.870	0.000	0.000	0.000	0.000	11846.32	2	180.95	0.000	0.000	0.000	0.000
	Hip circumference (cm)	12037.484	2	137.922	0.000	0.000	0.000	0.000	8035.28	2	120.53	0.000	0.000	0.000	0.000
	Waist-to-hip ratio	.325	2	33.795	0.000	0.001	0.000	0.000	0.17	2	28.60	0.000	0.309	0.000	0.000
	Waist-to-height ratio	.683	2	58.074	0.000	0.000	0.000	0.000	0.43	2	157.37	0.000	0.000	0.000	0.000
Adiposity Index															
	VAI	488.321	2	9.411	0.000	0.010	0.000	0.102	243.34	2	29.22	0.000	0.060	0.000	0.000
	LAP	89012.410	2	30.745	0.000	0.001	0.000	0.000	42599.66	2	47.51	0.023	0.000	0.000	0.000
Body fat mass – DXA															
	Total_FM_ (g)	9392648508.11	2	169.00	0.000	0.000	0.000	0.000	6200881733.65	2	103.71	0.000	0.000	0.000	0.000
	Total_%FM_	1552.94	2	46.98	0.000	0.000	0.000	0.001	1563.45	2	24.08	0.000	0.000	0.000	0.026
	Trunk_FM_ (g)	3020896318.80	2	203.40	0.000	0.000	0.000	0.000	1505674086.95	2	115.49	0.000	0.000	0.000	0.000
	Trunk_%FM_	3141.14	2	73.95	0.000	0.000	0.000	0.000	2580.10	2	33.75	0.000	0.000	0.000	0.001
	Android_FM_ (g)	124997069.49	2	180.85	0.000	0.000	0.000	0.000	61841649.02	2	113.65	0.000	0.000	0.000	0.000
	Android_%FM_	3682.36	2	71.05	0.000	0.000	0.000	0.001	3644.30	2	38.55	0.000	0.000	0.000	0.003
	Gynoid_FM_ (g)	190764435.80	2	75.61	0.000	0.000	0.000	0.001	106491403.45	2	42.01	0.000	0.000	0.000	0.001
	Gynoid_%FM_	220.47	2	4.75	0.010	0.022	0.004	0.618	471.17	2	5.62	0.000	0.010	0.004	0.811
Fat mass index – DXA															
	FMI (kg/m^2^)	1474.45	2	233.72	0.000	0.000	0.000	0.000	807.97	2	102.33	0.000	0.000	0.000	0.000
	A/G_Ratio_	1.16	2	48.63	0.000	0.000	0.000	0.000	1.04	2	54.04	0.000	0.000	0.000	0.000
	Trunk/Leg_s%FA_T	1.07	2	35.26	0.000	0.000	0.000	0.060	0.64	2	24.86	0.000	0.028	0.000	0.000
	Trunk/Limb_FAT_	1.61	2	22.82	0.000	0.000	0.000	0.189	0.61	2	14.88	0.000	0.248	0.000	0.000

P < 0.05. PCOS: polycystic ovary syndrome; 1: normal BMI; 2: overweight; 3: obesity; BMI, body mass index; VAI: visceral adiposity index; LAP: lipid accumulation product; And: android; Gyn: gynoid; FMI: total fat mass index; A/G ratio: android/gynoid fat ratio; Trunk/Limb_FAT_: trunk/limb fat mass ratio; Trunk/Legs_%FAT_: trunk/legs fat percentage ratio.

### Generalized linear model multivariate

WC, WHR, WHtR, trunk FM, And_FM_, A/G_RATIO_, and Trunk/Legs_%FAT_ and Trunk/Limb_FAT_ were associated with PCOS. The other covariates (age, FAI, and HOMA) were associated with several markers of fat mass, except Gyn_FM_ to age and HOMA-IR, and HipC and Gyn_FM_ to FAI (Table 4).

**Table 4:** Generalized linear model (GLM) results for the variables significantly associated with incidence de PCOS, age, FAI and HOMA-IR

Variables		PCOS	*P* Value	Age	*P* Value	FAI	*P* Value	HOMA-IR	*P* Value
		β (95% CI)		β (95% CI)		β (95% CI)		β (95% CI	
Anthropometric measurements									
	BMI (kg/m^2^)	0.65 (-0.87-2.16)	0.40	0.23 (0.10-0.36)	0.00	0.13 (0.02-0.23)	0.02	1.22 (0.79-1.66)	0.00
	Waist circumference (cm)	5.97 (2.75-9.20)	0.00	0.59 (0.32-0.87)	0.00	0.35 (0.13-0.58)	0.00	2.93 (2.00-3.86)	0.00
	Hip circumference (cm)	0.85 (-2.22-3.93)	0.59	0.28 (0.02-0.54)	0.04	0.16 (-0.05-0.37)	0.14	1.21 (0.32-2.10)	0.01
	Waist-to-hip ratio	0.05 (0.03-0.07)	0.00	0.00 (0.00-0.01)	0.00	0.00 (0.00-0.00)	0.00	0.02 (0.01-0.02)	0.00
	Waist-to-height ratio	0.04 (0.02-0.06)	0.00	0.00 (0.00-0.01)	0.00	0.00 (0.00-0.00)	0.00	0.02 (0.01-0.02)	0.00
Adiposity Index									
	VAI	0.07 (-1.10-1.24)	0.90	0.12 (0.03-0.22)	0.01	0.22 (0.14-0.30)	0.00	1.03 (0.69-1.36)	0.00
	LAP	2.95 (-5.22-11.12)	0.48	1.42 (0.73-2.11)	0.00	1.62 (1.06-2.19)	0.00	10.59 (8.23-12.94)	0.00
Body fat mass – DXA									
	Trunk_FM_	1812.40 (495.49-3129.30)	0.01	164.09 (52.74-275.45)	0.00	125.91 (34.24-217.58)	0.01	982.01 (602.68-1361.35)	0.00
	Trunk_%FAT_	3.91 (1.96-5.87)	0.00	0.13 (-0.04-0.29)	0.13	0.11 (-0.03-0.24)	0.13	0.80 (0.23-1.36)	0.01
	Total_FM_	2071.96 (-672.78-4816.69)	0.14	254.68 (22.60-486.77)	0.03	200.71 (9.65-391.77)	0.04	1548.21 (757.60-2338.83)	0.00
	Total_%FAT_	2.29 (0.60-3.98)	0.01	0.04 (-0.10-0.19)	0.54	0.02 (-0.10-0.14)	0.75	0.41 (-0.08-0.90)	0.10
	Android_FM_	281.23 (9.25-553.22)	0.04	34.06 (11.06-57.06)	0.00	24.14 (5.20-43.07)	0.01	213.42 (135.08-291.77)	0.00
	Android_%FAT_	3.36 (1.14-5.58)	0.00	0.12 (-0.07-0.31)	0.22	0.11 (-0.04-0.27)	0.16	0.94 (0.30-1.57)	0.00
	Gynoid_FM_	309.43 (-163.95-782.82)	0.20	22.67 (-17.36-62.70)	0.27	19.88 (-13.08-52.83)	0.24	110.38 (-25.98-246.73)	0.11
	Gynoid_%FAT_	1.41 (-0.17-3.00)	0.08	-0.08 (-0.21-0.06)	0.26	-0.07 (-0.18-0.04)	0.20	-0.27 (-0.73-0.18)	0.24
Fat mass index – DXA									
	FMI	0.85 (-0.40-2.10)	0.18	0.12 (0.02-0.23)	0.02	0.13 (0.04-0.21)	0.00	0.52 (0.16-0.88)	0.00
	A/GRATIO	0.06 (0.02-0.09)	0.00	0.00 (0.00-0.01)	0.00	0.00 (0.00-0.01)	0.00	0.03 (0.02-0.04)	0.00
	Trunk/Legs_%FAT_	0.08 (0.04-0.11)	0.00	0.00 (0.00-0.01)	0.00	0.00 (0.00-0.01)	0.00	0.03 (0.02-0.04)	0.00
	Trunk/Limb_FAT_	0.11 (0.06-0.16)	0.00	0.01 (0.00-0.01)	0.00	0.01 (0.00-0.01)	0.00	0.04 (0.02-0.05)	0.00

P < 0.05. β: constant – interpretation of GLMs coefficient; PCOS: polycystic ovary syndrome; %: percentage; FM, fat mass; BMI: body mass index; VAI: visceral adiposity index; LAP: lipid accumulation product; And: android; Gyn: gynoid; FMI: total fat mass index; A/G ratio: android/gynoid fat ratio; Trunk/Limb_FAT_: trunk/limb fat mass ratio; Trunk/Legs_%FAT_: trunk/legs fat percentage ratio.

## DISCUSSION

The markers of whole-BFM and central obesity are important for estimating abdominal adiposity to better stratify the risk of PCOS in women. We observed that women with PCOS showed increased Total_FM_ and fat accumulation in the central region compared to women without PCOS matched by BMI. This difference appears to be more pronounced in women with normal BMI in both DXA and adiposity index. Except for HipC, anthropometric measurements were increased in women with PCOS, independent of BMI. In the body fat distribution and central obesity indicators, progressive and significant increases in BMI were observed within the groups. However, no differences were observed in the trunk-to-peripheral fat ratio and VAI in women with PCOS, from overweight to obese, and in women without PCOS from normal BMI to overweight.

Fat accumulation is high in patients with PCOS, as estimated using DXA (6,16,17). Carmina and cols. observed an increase in the amount of central abdominal fat in PCOS patients, but not in total and trunk fat. Moreover, only women who presented with increased central abdominal fat had higher insulin and androgen levels and reduced insulin sensitivity (6). Another trial showed that lean patients with PCOS had higher trunk/periphery fat ratios and reduced insulin sensitivity. However, the trunk/peripheral fat ratio in obese PCOS patients, despite significantly higher levels of total and free testosterone and reduced insulin sensitivity, did not differ from that in women with regular menstrual cycles (16). Satyaraddi and cols. observed that Total_FM_ and Trunk_FM_ were higher in PCOS women than in non-PCOS controls matched by BMI. Moreover, PCOS patients with and without obesity had increased visceral adiposity, with a difference even after correcting for body weight. Additionally, the IR was more prevalent in obese PCOS patients (80%) than in non-obese individuals (20%), as well as hyperandrogenism, either biochemical or clinical, 60% and 30%, respectively (17). Our results support these findings. Although we observed a high amount of trunk fat and trunk/periphery fat ratios in obese women with PCOS compared to their BMI-matched controls, obesity was not able to modify these ratios in PCOS from overweight to higher BMI. This and the similarity of the other DXA indicators among obese women suggests that obesity is not a factor that significantly increases the accumulation of total and abdominal fat in PCOS, but being overweight does. Most individuals, regardless of having PCOS, have abdominal obesity.

In this study, HOMA and FAI, along with the age and incidence of PCOS, were predictors of body fat distribution and central obesity by DXA, except for gluteus-femoral (i.e., gynoid) fat mass. Gynoid fat mass and percentage of gynoid fat relative to the total body were similar in overweight and obese women. However, in women with normal BMI, the percentage of gynoid fat was high in those with PCOS. In contrast, android fat mass, percent android fat relative to total body fat, and A/G ratio were similar between obese women. Dumesic and cols. showed a similarity in the fat of the gynoid region and an increase in the fat of the android region. They also reported that neither GynFM nor percent gynoid fat relative to total body fat were related to circulating androgen or insulin levels. However, intra-abdominal fat deposition is positively correlated with insulin levels and hyperandrogenism in PCOS (18) i.e., hyperandrogenism, independent of BMI and HOMA in women with PCOS, is associated with preferential deposition of fat in the abdominal region, linking visceral fat, hyperinsulinemia, and hyperandrogenism.

A higher BMI in PCOS is associated with VAI and LAP obesity indices (19-21). In our study, LAP and VAI showed worse scores from normal BMI to obese women with PCOS, and the same curve of these indices was observed in women without PCOS. However, VAI was not different in women with PCOS from overweight to obese and in women without PCOS from lean to overweight. Although the explanations for this need to be clarified, it is likely that the changes associated with triglycerides may be reflected in the VAI. The VAI is a calculated model based on a combination of anthropometric and laboratory data. Moreover, in this study, women with PCOS and normal BMI were found to have higher VAI and LAP values than women without PCOS. This was not observed in the overweight and obese female subgroups. LAP and VAI are effective markers for stratifying metabolically obese normal-weight adults and are more likely to accumulate visceral fat, regardless of sex and age. These indices are strongly related to this phenotype, with the supremacy of VAI over LAP, regardless of the criteria used to define the phenotype (22).

Age, HOMA, and FAI were related to LAP and VAI indices, but not to the incidence of PCOS. A previous study showed that lean PCOS and RI had higher values for LAP and VAI concerning lean PCOS without RI. This study also showed that both LAP and VAI may be useful for the assessment of hyperandrogenism in PCOS lean (23) and predicting IR in women with normal weight (24). For overweight/obese women with PCOS, anthropometric indices, such as BMI and WHtR, are more effective in predicting IR (24). However, a recent study demonstrated the superiority of VAI in predicting metabolic syndrome and RI in women with PCOS with BMI < 30 and BMI ≥ 30 over other anthropometric parameters or anthropometric-metabolic indices (21).

We observed that some anthropometric parameters, such as WC, WHtR, and WHR, except HipC, showed visceral adiposity accumulation to some degree among women with PCOS when compared with their BMI-matched controls. The higher the BMI, the worse the scores of these parameters in both groups, women with PCOS and non-PCOS. Our results are similar to those of other studies (19,25). However, Chitme and cols. observed a higher percentage of women with PCOS with large and extra-large circumference hip, and that increased WC and HipC were associated with an increased incidence of PCOS (26). In our study, the incidence of PCOS was associated with WC, WHtR, and WHR, but not with HipC and BMI. Age, HOMA, and FAI were predictors of WC, WHtR, and WHR, except for the FAI for HipC.

Among the anthropometric indices mentioned previously, BMI stands out. BMI lacks discriminatory power between fat and lean tissues for a standardized definition of obesity (27), i.e., there was no linear association with body fat percentage (28). Although several women with PCOS have a normal body fat level based on BMI, our results corroborate those of a previous study (29) and demonstrate that most women with PCOS and normal BMI have an excess body fat level. Moreover, body fat percentage, but not BMI, is a better marker for measuring inflammation related to body fat accumulation in PCOS (30). We observed that the incidence of PCOS was a predictor of body fat percentage, but not BMI. Based on BMI, some studies classify PCOS into obese-overweight and normal BMI phenotypes (4,31) and suggest that there may be different metabolic profiles between these phenotypes because of different proportions of adipose tissue (24,31). Our study also found that HOMA-IR value increased with weight gain and fasting insulin regardless of PCOS, and high values of fasting glycemia in line with the higher BMI in PCOS.

The comparison of lipid profiles in our sample based on BMI showed similarity in mean levels of LDL, TG, TC, and HDL. Contrastingly, other studies have shown that lipid profiles in obese PCOS and non-obese PCOS patients demonstrated significant differences in levels of TC, TG, and LDL compared to their BMI-matched controls (32). However, conflicting results can be found in the study population due to factors such as race, age, genetics, diet, lifestyle, and differences, in economics. However, dyslipidemia in PCOS occurs within a set of interrelated pathological variables (33). In our study, while investigating the relationship between metabolic issues, hyperandrogenism, and BMI, we found that while in PCOS, the presence of overweight worsens TG and HDL scores, in women without PCOS these changes occur only in the presence of obesity and in LDL in PCOS. This demonstrates that dyslipidemia is associated with obesity, independent of PCOS.

In all the subgroups, women with PCOS had higher testosterone and fasting insulin levels and FAI and HOMA scores. Luotola and cols. found that in women with PCOS, testosterone levels and increased FAI values are associated with a reduction in the insulin sensitivity index and an increase in early insulin secretion regardless of adiposity, i.e., elevated testosterone levels, even within the normal range, can alter insulin sensitivity in women with PCOS (5). Additionally, the HOMA-IR value increased with weight gain and fasting insulin in both groups. In women with PCOS, the FAI increased directly proportional to BMI, with a greater value in obese women, and SHBG showed an inversely proportional increase in BMI, with a greater value in the normal BMI group. In non-PCOS women, the difference was only from overweight to obese. Moreover, the SHBG levels decreased as obesity indicators increased. In obese individuals, the androgenic transition accelerates depending on the SHBG reduction, and androgenic synthesis increases to correspond to this (34). SHBG is responsible for regulating the biological activities of sex hormones and the main transporter proteins of estradiol and testosterone (35), affecting their bioavailability (36).

The presence of excess androgens can characterize the development of obesity, especially visceral adiposity, during adolescence and adulthood. This may favor the development of metabolic disorders at any age, as a condition of PCOS secondary to obesity in adolescents and women of reproductive age (37). There is reportedly a 36.6% increase in the risk of developing PCOS in women with higher total body fat (26). However, the mechanisms mediating this process are more complex than a simple cause-and-effect process (38).

The strength of this study was that PCOS patients were carefully characterized for clinical trials. Moreover, the groups evaluated were paired for BMI, thus reducing the confounding effects of this variable. Although we collected details on sedentary lifestyle in the PCOS and non-PCOS groups, this study was limited by the retrospective nature of a convenience sample. Detailed studies clearly defining body composition by assessing visceral, subcutaneous, central, and peripheral fat mass may provide more reliable information on the evolution of body fat distribution following BMI and phenotypes.

In conclusion, in women with PCOS and normal BMI, DXA and the adiposity indices, VAI and LAP, are more sensitive methods to evaluate total body fat and fat accumulation in the central abdominal region. A higher BMI was found to cause specific problems of PCOS and may be associated with similar comorbidities in women of reproductive age, independent of PCOS. Moreover, IR was associated with PCOS, regardless of the BMI. However, overweight adversely affected many aspects of PCOS, such as metabolic abnormalities, while obesity further worsened these outcomes. These findings aid understanding of the complexity of PCOS, demonstrating the distribution of body fat, and hormonal and metabolic profiles of women matched by BMI. The use of viable methods, such as VAI and LAP, to estimate central obesity, especially in thin women, can assist in clinical practice.

## References

[1] Lizneva D, Suturina L, Walker W, Brakta S, Gavrilova-Jordan L, Azziz R (2016). Criteria, prevalence, and phenotypes of polycystic ovary syndrome. Fertil Steril.

[2] Teede H, Deeks A, Moran L (2010). Polycystic ovary syndrome: A complex condition with psychological, reproductive and metabolic manifestations that impacts on health across the lifespan. BMC Med.

[3] Romano LGM, Bedoschi G, Melo AS, Albuquerque FO, Silva ACJ, Ferriani RA (2011). Anormalidades metabólicas em mulheres com síndrome dos ovários policísticos: obesas e não obesas. Rev Bras Ginecol Obstet.

[4] Toosy S, Sodi R, Pappachan JM (2018). Lean polycystic ovary syndrome (PCOS): an evidence-based practical approach. J Diabetes Metab Disord.

[5] Luotola K, Piltonen TT, Puurunen J, Morin-Papunen LC, Tapanainen JS (2018). Testosterone is associated with insulin resistance index independently of adiposity in women with polycystic ovary syndrome. Gynecol Endocrinol.

[6] Carmina E, Bucchieri S, Esposito A, Del Puente A, Mansueto P, Orio F (2007). Abdominal fat quantity and distribution in women with polycystic ovary syndrome and extent of its relation to insulin resistance. J Clin Endocrinol Metab.

[7] Albanese CV, Diessel E, Genant HK (2003). Clinical applications of body composition measurements using DXA. J Clin Densitom.

[8] Almeida RT, Almeida MM, Araújo TM (2009). Abdominal obesity and cardiovascular risk: performance of anthropometric indexes in women. Arq Bras Cardiol.

[9] Kahn HS (2005). The “lipid accumulation product” performs better than the body mass index for recognizing cardiovascular risk: A population-based comparison. BMC Cardiovasc Disord.

[10] Amato MC, Giordano C, Galia M, Criscimanna A, Vitabile S, Midiri M (2010). Visceral adiposity index: A reliable indicator of visceral fat function associated with cardiometabolic risk. Diabetes Care.

[11] Rotterdam ESHRE/ASRM-Sponsored PCOS Consensus Workshop Group (2004). Revised 2003 consensus on diagnostic criteria and long-term health risks related to polycystic ovary syndrome. Fertil Steril.

[12] Geloneze B, Repetto EM, Geloneze SR, Tambascia MA, Ermetice MN (2006). The threshold value for insulin resistance (HOMA-IR) in an admixtured population. IR in the Brazilian Metabolic Syndrome Study. Diabetes Res Clin Pract.

[13] Friedewald WT, Levy RI, Fredrickson DS (1972). Estimation of the concentration of low-density lipoprotein cholesterol in plasma, without use of the preparative ultracentrifuge. Clin Chem.

[14] Cascella T, Palomba S, Tauchmanovà L, Manguso F, Di Biase S, Labella D (2006). Serum aldosterone concentration and cardiovascular risk in women with polycystic ovarian syndrome. J Clin Endocrinol Metab.

[15] Biyik Z, Guney I (2019). Lipid accumulation product and visceral adiposity ındex: two new indices to predict metabolic syndrome in chronic kidney disease. Eur Rev Med Pharmacol Sci.

[16] Svendsen P, Nilas L, Norgaard K, Jensen JE, Madsbad S (2008). Obesity, body composition and metabolic disturbances in polycystic ovary syndrome. Hum Reprod.

[17] Satyaraddi A, Cherian K, Kapoor N, Kunjummen A, Kamath M, Thomas N (2019). Body composition, metabolic characteristics, and insulin resistance in obese and nonobese women with polycystic ovary syndrome. J Hum Reprod Sci.

[18] Dumesic DA, Akopians AL, Madrigal VK, Ramirez E, Margolis DJ, Sarma MK (2016). Hyperandrogenism accompanies increased intra-abdominal fat storage in normal weight polycystic ovary syndrome women. J Clin Endocrinol Metab.

[19] Ribeiro VB, Kogure GS, Lopes IP, Silva RC, Pedroso DCC, Ferriani RA (2019). Association of measures of central fat accumulation indices with body fat distribution and metabolic, hormonal, and inflammatory parameters in women with polycystic ovary syndrome. Arch Endocrinol Metab.

[20] Durmus U, Duran C, Ecirli S (2017). Visceral adiposity index levels in overweight and/or obese, and non-obese patients with polycystic ovary syndrome and its relationship with metabolic and inflammatory parameters. J Endocrinol Invest.

[21] de Medeiros SF, de Medeiros MAS, Barbosa BB, Yamamoto MMW (2021). The Role of Visceral Adiposity Index as Predictor of Metabolic Syndrome in Obese and Nonobese Women with Polycystic Ovary Syndrome. Metab Syndr Relat Disord.

[22] Du T, Yu X, Zhang J, Sun X (2015). Lipid accumulation product and visceral adiposity index are effective markers for identifying the metabolically obese normal-weight phenotype. Acta Diabetol.

[23] Anik Ilhan G, Yildizhan B, Pekin T (2019). The impact of lipid accumulation product (LAP) and visceral adiposity index (VAI) on clinical, hormonal and metabolic parameters in lean women with polycystic ovary syndrome. Gynecol Endocrinol.

[24] Huang X, Wang Q, Liu T, Pei T, Liu D, Zhu H (2019). Body fat indices as effective predictors of insulin resistance in obese/non-obese polycystic ovary syndrome women in the Southwest of China. Endocrine.

[25] Kogure GS, Miranda-Furtado CL, Pedroso DCC, Ribeiro VB, Eiras MC, Silva RC (2019). Effects of progressive resistance training on obesity indices in polycystic ovary syndrome and the relationship with telomere length. J Phys Act Health.

[26] Chitme HR, Al Azawi EAK, Al Abri AM, Al Busaidi BM, Salam ZKA, Al Taie MM (2017). Anthropometric and body composition analysis of infertile women with polycystic ovary syndrome. J Taibah Univ Med Sci.

[27] Gallagher D, Heymsfield SB, Heo M, Jebb SA, Murgatroyd PR, Sakamoto Y (2000). Healthy percentage body fat ranges: An approach for developing guidelines based on body mass index. Am J Clin Nutr.

[28] Wildman RP (2008). The Obese Without Cardiometabolic Risk Factor Clustering and the Normal Weight With Cardiometabolic Risk Factor Clustering. Arch Intern Med.

[29] Rojas J, Chávez M, Olivar L, Rojas M, Morillo J, Mejías J (2014). Polycystic Ovary Syndrome, Insulin Resistance, and Obesity: Navigating the Pathophysiologic Labyrinth. Int J Reprod Med.

[30] Hestiantoro A, Hasani RDK, Shadrina A, Situmorang H, Ilma N, Muharam R (2018). Body fat percentage is a better marker than body mass index for determining inflammation status in polycystic ovary syndrome. Int J Reprod Biomed.

[31] Sachdeva G, Gainder S, Suri V, Sachdeva N, Chopra S (2019). Obese and non-obese polycystic ovarian syndrome: Comparison of clinical, metabolic, hormonal parameters, and their differential response to clomiphene. Indian J Endocrinol Metab.

[32] Halasawadekar N, Ramanand J, Ramanand S, Raparti G, Patil P, Shah R (2016). Serum lipid profile in non-polycystic ovary syndrome and polycystic ovary syndrome women: a comparative and correlational study. Int J Basic Clin Pharmacol.

[33] Liu Q, Xie YJ, Qu LH, Zhang MX, Mo ZC (2019). Dyslipidemia involvement in the development of polycystic ovary syndrome. Taiwan J Obstet Gynecol.

[34] Sayln S, Kutlu R, Kulakslzoğlu M (2020). The relationship between sex steroids, insulin resistance and body compositions in obese women: A case-control study. J Med Biochem.

[35] Dunn JF, Nisula BC, Rodbard D (1981). Transport of steroid hormones: Binding of 21 endogenous steroids to both testosterone-binding globulin and corticosteroid-binding globulin in human plasma. J Clin Endocrinol Metab.

[36] Xing C, Zhang J, Zhao H, He B (2022). Effect of Sex Hormone-Binding Globulin on Polycystic Ovary Syndrome: Mechanisms, Manifestations, Genetics, and Treatment. Int J Womens Health.

[37] Pasquali R, Oriolo C (2019). Obesity and Androgens in Women. Front Horm Res.

[38] Barber TM, Hanson P, Weickert MO, Franks S (2019). Obesity and Polycystic Ovary Syndrome: Implications for Pathogenesis and Novel Management Strategies. Clin Med Insights Reprod Health.

